# Lived Experiences of Tooth Hypersensitivity in Children With Molar Incisor Hypomineralisation

**DOI:** 10.1111/ipd.70093

**Published:** 2026-05-01

**Authors:** Joana Monteiro, Fiona Gilchrist, Helen Rodd

**Affiliations:** ^1^ University of Sheffield Sheffield UK

**Keywords:** disturbances in dental development, growth and development, pain control, sedation

## Abstract

**Background:**

Children with molar incisor hypomineralisation (MIH) can suffer from intense tooth hypersensitivity (TH), which may adversely affect various aspects of their emotional, social, and functional wellbeing. As TH diagnostic tools are primarily adapted from adult research, there is a clear need for a deeper understanding of MIH‐TH from a child‐centred perspective.

**Aim:**

To explore how children with MIH perceive and experience TH within the context of their daily lives.

**Design:**

Following ethical approval, audio‐recorded, semi‐structured interviews were conducted with children with MIH, aged between 6‐ and 16‐years‐old, followed by iterative thematic analysis.

**Results:**

Seventeen children were included (10 girls and 7 boys) with a mean age of 9 years and 7 months. All children had severe MIH, with between 1 and 10 affected teeth. Five main themes emerged: description of TH, triggers for TH, impact of TH, responses to TH, and *experience within the dental context and setting*. Children used rich language to convey their experiences of TH and its various impacts.

**Conclusion:**

Children with MIH described their experiences of TH, highlighting its considerable impact on their daily lives. Data from this study will inform the development of a TH‐specific measurement tool for this patient group.

## Why This Paper Is Important to Pediatric Dentists

1. Children with MIH experience tooth hypersensitivity (TH) that significantly impacts on their quality of life.
2. Children may develop different strategies to cope with TH, ranging from acceptance to active problem‐solving.
3. Anticipated or actual pain in the dental setting was a common concern.

This paper highlights the need for healthcare professionals to consider TH and its profound impact on children with MIH. Communication is key and valued by this patient group.

## 1 Introduction

Molar incisor hypomineralisation (MIH) is a common enamel disorder, affecting 13% of children globally [1, 2]. Environmental and genetic factors are thought to interact, resulting in developmental enamel defects predominantly in first permanent molars and incisors. Due to suboptimal biochemical and mechanical characteristics, affected enamel can be soft, porous and prone to breaking down, leading to pain and aesthetic concerns. Notably, children with MIH can experience extreme tooth hypersensitivity (TH), which may impact negatively on their oral health‐related quality of life (OHRQoL) [3, 4, 5].

Although TH is commonly observed in children with MIH, it remains poorly understood with sparse supporting research [6]. To date, tests used to diagnose TH have largely been adapted from investigations with adults, often involving pain‐inducing stimuli and generic questionnaires, which are not designed to assess responses in children with enamel defects [7]. It is thus impossible to know the extent and impact of TH on MIH‐affected children, or the effectiveness of any interventions, potentially leaving them with ongoing symptoms. It is crucial to explore TH from the child's perspective, understanding the words they use to describe it, their experience of triggers, and the ways in which it affects their daily lives, social interactions, and emotional wellbeing. Understanding children's own language and lived experiences helps reveal how TH is recognised, expressed, and managed. These insights may be missed by quantitative measures or by tools designed for adults, especially when symptoms are intermittent or not visibly apparent. Qualitative enquiry helps to gain insight into how and to whom children communicate their symptoms, how they experience the dental setting, and how this may influence their dental care. Understanding these dimensions can support communication, inform tailored clinical interventions, and ensure that dental management addresses not only the physical aspects but also the psychosocial impact of this condition.

There is, therefore, a real need to better understand the context of MIH‐related TH from the child's perspective. For this reason, the aim of this study was to use qualitative and child‐centred research methodology to gain greater insight into how children with MIH experience and understand TH in the context of their everyday lives. This paper represents the foundational exploratory phase of a wider project to develop and evaluate a child OHRQoL measure of TH specific to children with MIH. By providing novel and meaningful insights into children's own language, perceptions, and lived experiences of TH, this study ensures that the tool will be grounded in children's perspectives from the outset. Children's involvement is central to the whole project, with engagement planned at every stage, through patient and public involvement (PPI), piloting, cognitive interviews, validation, and children engagement in dissemination of findings.

## 2 Materials and Methods

This study sought to include children aged 6‐ to 16‐years‐old referred to the paediatric dentistry clinic for the management of their MIH, following ethical approval by the South Central‐Oxford C Research Ethics Committee in November 2023 (REC 23/SC/0368; IRAS: 333349). Written parental/carer consent and child assent were obtained prior to participation, and families were given the option of face‐to‐face or online interviews. No ethical concerns arose during this project.

### 2.1 Study Design

A qualitative semi‐structured interview design, supported by a topic guide, was employed to broadly explore children's experiences of MIH and associated TH. Thematic analysis was undertaken following the methodology described by Braun and Clarke [8]. Thematic analysis was inductive and involved the following stages: identifying initial themes; labelling the data; sorting the data by themes and synthesising the data.

All the researchers were paediatric dentists. Two have extensive experience in qualitative research, and the principal investigator (PI) undertook recognised training in qualitative research methods (provided by the UK National Centre for Social Research). The classification and severity of MIH was determined in each participant by the PI (JM) following training and calibration using a validated MIH index [9, 10]. Cohen Kappa coefficient scores were found to be between 0.71 and 0.94, demonstrating substantial and almost perfect agreement, respectively.

### 2.2 Participants

Families were purposively approached by a member of the research team and given information leaflets which had been designed in collaboration with five children. Inclusion criteria encompassed children with MIH, diagnosed following clinical and radiographic assessments and who spoke and read English. Exclusion criteria were: children who had enamel defects that were not typical of MIH; other conditions that might be associated with dental pain or hypersensitivity; pre‐existing medical conditions that were ASA Grade 3 or greater or severe learning difficulties [11]. All children were examined by a member of the research team and completed their course of treatment as planned. Recruitment continued, alongside contemporaneous data analysis of interview transcripts until the data were judged to be sufficiently rich to reflect the perspectives of diverse participants.

### 2.3 Interviews

An interview topic guide was developed from the existing MIH and TH literature and guided by the Wilson and Cleary theoretical model of health‐related quality of life [12]. Questions related to the presence/absence of TH, children's descriptions of this condition, and the impact and experience of any interventions to manage TH. Additionally, visual prompts depicting daily activities identified in the literature as potential triggers for TH were employed to facilitate discussion. Children were invited to choose a pseudonym to preserve their anonymity throughout the study. Participants were able to decide whether they would like their parents/carers to be present, but the questions were directed towards children themselves. The interviews were audio‐recorded and transcribed verbatim by a private company (Dictate2us Limited). Interviews were exploratory, consisting of open‐ended questions, based on the topic guide, with freedom to follow particular lines of discussion as it felt appropriate. It was important to ensure flexibility for the introduction of topics of relevance to participants, regardless of whether they were initially included in the topic guide. This allowed the emergence of new topics and wording, which were iteratively incorporated into subsequent interviews.

### 2.4 Data Analysis

All audio records were analysed using qualitative data analysis software (NVivo 12, QSR International, Melbourne, Australia). Thematic analysis was conducted concurrently with data collection until no new themes emerged. The first three interviews were transcribed and coded by all three investigators. Along with field notes and memos, this process facilitated a deeper familiarity with the data. From the initial codes, topics related to TH were identified. By identifying patterns and connections, topics were organised into distinct themes, ensuring that each theme was grounded in the data. Recurrent themes were grouped and organised into an initial thematic framework. Following the indexing of all transcripts individually, the 17 interviews were analysed as a whole, allowing for further refinement. This phase involved collapsing candidate themes together, splitting them into new themes, or discarding some themes. Finally, notes were discussed for the whole dataset, and disagreements were resolved. The generated initial themes were reviewed to ensure they were coherent with coded extracts and reflected the full dataset.

## 3 Results

**FIGURE 1:**
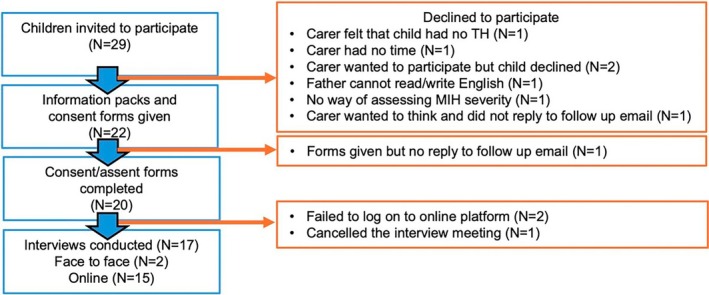
Participant flowchart.

A total of 29 parent/child dyads were approached. Nine parent/carer pairs declined and 20 agreed to participate, giving a recruitment rate of 68%. Following consent/assent, two carers did not log onto the online platform, and one cancelled their interview with an overall response rate of 59% (see Figure 1).

Children were aged between 6‐ and 15‐years, with a similar number of male and female participants and good representation from all deprivation quintiles. Most participants were from a Northern European background, with one patient identifying as being of Black African descent. One patient had autism, with the remaining participants having no relevant medical histories. All patients had a diagnosis of severe MIH according to the Academy of Paediatric Dentistry's classification [10], with the number of affected teeth ranging between 1 and 10 (Table 1).

**TABLE 1:** Demographic information.

Patient identifier	Sex	Ethnicity	Multiple deprivation decile	Age at assessment	Number of affected teeth	Additional findings	Overall MIH severity
C1‐1	Male	White	9	10 year 1 month	6	Cavitation 46, crossbite	Severe
C1‐2	Female	White	5	15 year 10 month	10		Severe
C1‐3	Female	White	8	8 year 9 month	2	Caries ULD not into pulp	Severe
C1‐4 did not log in to interview							
C1‐5	Male	White	7	11 year 0 month	3		Severe
C1‐6	Female	White	10	9 year 1 month	3	Caries URD, URE, ULD, ULE	Severe
C1‐7	Male	White	6	12 year 11 month	4		Severe
C1‐8	Male	White	10	9 year 5 month	8	Es are affected, taurodontism FPM	Severe
C1‐9 did not log in to interview	Male	White	4	7 year 5 month	5	Hypodontia missing LR1, LL1	Severe
c1‐10	Female	White	9	7 year 6 month	7		Severe
c1‐11	Male	White	7	11 year 9 month	4	PMC autistic	Severe
c1‐12	Male	White	4	9 year 1 month	5		Severe
c1‐13	Female	White	7	6 year 6 month	4		Severe
c1‐14	Male	White	5	8 year 2 month	1		Severe
c1‐15—cancelled interview	Male	White	5	7 year 2 month			
c1‐16	Female	White	8	8 year 10 month	7	Nad	Severe
W1‐1	Female	White	4	10 year 0 month	2	Nad	Severe
W1‐2	Female	White	1	9 year 6 month	4		Severe
c1‐17	Female	Black	2	8 year 6 month	6	Nad	Severe
w1‐3	Female	White	1	6 year 2 month	6		Severe

Interviews were held with 17 children, between December 2023 and February 2024. The length of the interviews ranged from around three to 26 min, with a mean duration of approximately 14 min. The preferred approach for interviews was online, with 15 participant/carer pairs choosing this option. Most parents were present during the interviews, with one older child logging in unaccompanied.

**FIGURE 2:**
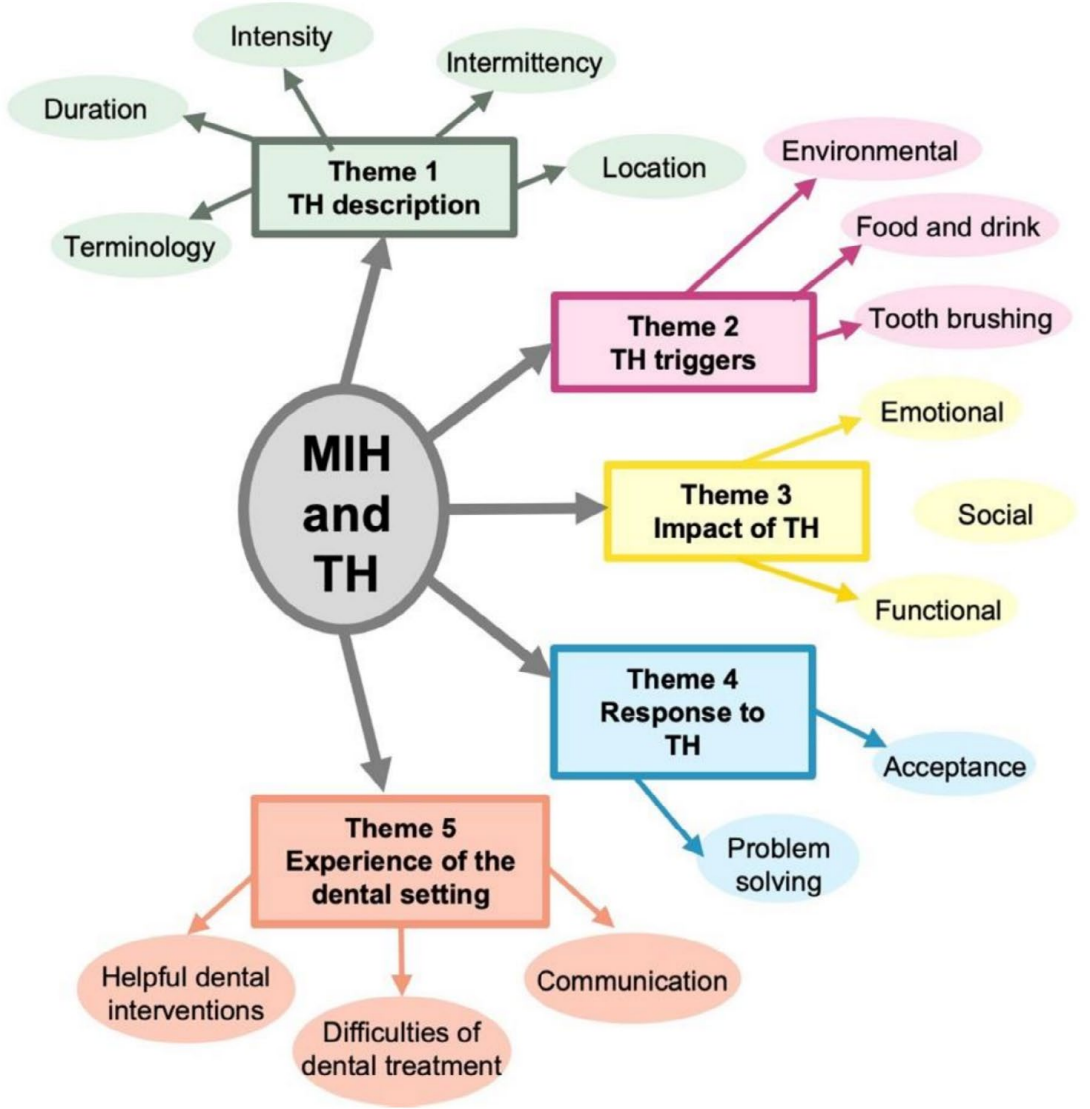
Coding tree showing tooth hypersensitivity themes and sub‐themes from interviews with 17 children with molar incisor hypomineralisation.

Following analysis, five themes were identified: (1) TH description; (2) TH triggers; (3) impact of TH; (4) response to TH and (5) experience within the dental context and setting. Sub‐themes were identified for each of the main themes as shown in Figure 2.

### 3.1 Theme 1: TH Description

The first theme encompassed five subthemes: terminology used by children to describe symptoms related to TH; duration of symptoms; intensity of symptoms (referring to the length of time each individual episode of pain persisted); location and intermittency of TH. When asked to describe the feeling of TH, children used a variety of words, facial/body expressions and sounds, as illustrated in the following extracts.

It feels like something's banging on it. Anna, aged 8 years

When you have sensitive teeth, or bad cold on your teeth you can't see. This is, like, my face… how do you describe… [making a grimace] … So, yeah, I grimaced when that happened, so it's not very nice, really Nitro, male, aged 8 years

When asked to describe the duration of each discrete episode of TH, some children found it difficult to quantify, whereas others were very specific about their time recollection. A wide variety of time frames were discussed, from a couple of seconds to 15 min.

It depends like how fast you swallow it, so probably for me it'd be about 2–3 s. Emily, aged 10 years old

I would say about 5–10 min. Livy, aged 9 years

When asked to describe TH intensity, children used words such as “bad” and compared it to other bodily pains. When this happened, TH was perceived as being worse than a tummy ache or a knee scratch.

Because scratching [a knee] it is like, it does hurt, but it's not really that painful. So, I'd say that my toothache is more painful than that. Eric, aged 11 years.

Children described TH as “7 out of 10” on a pain scale, recalled needing analgesics or even screaming and crying due to TH. One child with autism, who communicated with gestures and simple language, said it felt *“bad”* and put his thumbs down (Luke, aged 11 years).

If I was saying like 10, I'd probably be saying like 7. Anna, aged 8 years

Children described the intermittent nature of TH in their own words, without prompting. The wording used included “not all the time” or “on and off”. Children often felt that the posterior teeth were more affected by TH.

Not all the time. It's just on and off. Jeff, aged 10 years

Sometimes, it doesn't feel weird as much and sometimes it does. Lucy, aged 8 years.

### 3.2 Theme 2: TH Triggers

When children were asked which activities triggered TH, several sub‐themes emerged including food and drink, tooth brushing, and environmental triggers. The main trigger for TH seemed to be cold food or drinks, but hot (very warm) food and drinks were also described as triggers. Other triggers included sugary or chewy foods or carbonated drinks.

…that's why I don't really like ice cream that much… Because it gives me goose bumps and like always happens. Jeff, aged 10 years

I used to not be able to eat strawberries with my back teeth. Emily, female, aged 10 years old

When discussing some fruit, sour foods and drinks, children attributed TH to the cold temperature or hardness rather than acidity.

Apple is fine…sometimes it's a bit like the juice is sour. But apples are fine. Gerald, aged 12 years

Some children reported that tooth brushing with cold toothpaste caused TH. Despite disclosing feelings of discomfort when brushing their teeth, several children acknowledged the importance of maintaining their oral hygiene. This seemed to become more relevant as children grew older.

It's stopped now, but like before when I brushed my teeth, it had like a tingly feeling and it just kind of hurt a bit. And it was like the sharp pain as well, when I had to brush over it, I would try and avoid it a bit…Now I know that if it hurt a bit, I would still have to brush it even if it hurt, because otherwise then it would get a bit rotten. Gerald, aged 12 years

Children also reported cold air as a trigger, when speaking or playing outside in cold weather.

It will only hurt if…I was talking and it was snowing, it would kind of hurt but if I wasn't talking and it was snowing, it wouldn't hurt that much. Ella, aged 9 years.

### 3.3 Theme 3: Impact of TH

Several children discussed the impacts of TH in their daily lives. This theme was found to include three subthemes: functional, social, and emotional impacts of TH.

Participants discussed the functional impacts of TH on their food or drink consumption:

I have my packed lunch, but my tuna sandwich is usually a bit cold, so it could sometimes, [cause TH]…I also have an apple, sometimes I bite it on that side, forget about my tooth, and then it starts getting a bit sensitive because the apple's cold. Emily, aged 10 years old

Children discussed the impact that TH had on activities with friends. At times TH would make them stop “having fun with friends”. Some children discussed it openly with their friends, while others tried not to show they had TH.

Let's say if I am having a 100% fun, and then if I have sensitivity, it just goes down by 10%…maybe 15%, depending how much sensitivity I have; I said ruins it by 10–15, I still don't like it (laughs) either way. Nitro, aged 8 years

In addition to functional and social impacts, some participants described how TH affected them, emotionally. Children said it made them feel weaker, that they felt misunderstood and thought they were the only affected people, especially when they were younger. Some children discussed how they felt frustrated about TH or viewed it as a ‘looming fear’.

It makes me feel a little bit weaker…a bit anxious. Ella, aged 9 years

It would just make me a bit upset and then you get frustrated about the pain and your teeth are hurting and then you get agitated and then everything just starts to go wrong, you know. I guess there's always that looming fear that you're going to have the pain and it's going to ruin…especially if you're out with your friends, it's going to ruin a special moment…you're waiting for it, because you know it's going to inevitably happen with something colder. Maggie, aged 15 years.

Children discussed that TH was unlike other bodily pains as it could not be seen.

No, [like] nothing else that I've had in my body…I don't really compare, it's just like, when you get a scratch on your knee, pain, right? But then when you have sensitive teeth, or bad cold on your teeth, whatever…you can't see. Nitro, aged 8 years

It's, it was kind of like a…not a like a stinging, but like an internal, kind of numbing pain. It's really hard to explain in words, it like a weird pain, like know there's a pain there, but because it's inside your teeth, it's weird because it's not like, you know…a cut on your hand or something. You can't physically see it. Maggie, aged 15 years.

When asked to discuss the impact of this invisible pain, children referred to the other people's perception, including parents.

…with parents and stuff when you're like, oh my teeth hurt because of these certain things. They're like, what do you mean your teeth hurt? …You should be fuming like that, but when you're younger you're just like…they hurt all around and everything, maybe it's the gums but like you know that it is my teeth. Maggie, aged 15 years.

Some children discussed it openly with their friends, while others tried not to show they had TH.

So, with my friends, I'm just like, I grimace but I don't really show it, really, because I'm like, why do they need to know? Nitro, aged 8 years.

### 3.4 Theme 4: Response to TH

The way children coped with TH was discussed by several participants. Sub‐themes included problem‐solving and acceptance. Younger children discussed that distraction could help to reduce TH.

When I'm doing like something with my toys and my sisters it goes away. (Rose, aged 6 years)

At times, children avoided certain foods or drinks, or they tried to avoid affected teeth when they consumed TH‐inducing items.

…cold drinks when they're really cold and you just don't want to drink them all. Maggie, aged 15 years

Several children discussed their coping strategies including avoidance of certain teeth or areas in the mouth, eating with non‐affected teeth (chewing on the other side), and ensuring cold or hot drinks and foods did not touch sensitive teeth.

Hot drinks are fine; you just have to try and make it that it doesn't touch the back teeth. But when it does, it feels a bit, you know, like, icky, yeah. Gerald, aged 12 years

Participants described coping mechanisms that included problem‐solving, such as adjustments when eating and drinking, and emotional adaptation as they got older. Some children discussed that stopping eating/drinking would help resolve TH. Physical and functional adaptations included waiting for frozen food items to melt or eating differently to avoid TH.

…with ice cream, I eat it, but I…somehow protect my teeth with my tongue like cover it. Emily, aged 10 years old

if it's cold [air] there's not really anything you could do other than maybe covering your mouth like you know, with a scarf or something…because you just breathe in your own warm air. Maggie, female, aged 15 years

Children also referred to adaptations when brushing their teeth:

…in my house it takes a while for the water to become warm through the tap…so I…just quickly brush my teeth, do it as fast as possible which probably isn't the best for your teeth, but when they hurt, you do what you can. Maggie, aged 15 years

…my toothpaste sometimes can be really cold because it's been left out, so…I usually start with my tongue and work my way up. But sometimes if I don't start with my tongue, it's a bit sensitive on them teeth. Emily, aged 10 years

Children discussed emotional self‐regulation when TH was experienced.

I would just try getting it out of my mind. Ella, aged 9 years

Young people discussed how they learned to accept TH as they felt they understood their condition better as they grew older.

…but like it's stopped now, so it's like calmed down a bit and because I was only young, so I didn't really know that it would hurt…growing and maybe just getting used to it a bit more…well, I'm not as sensitive to it as I was when I was younger, I understand it more now, I guess. Gerald, aged 12 years

I've learned to adapt with my two sensitive teeth. Emily, aged 10 years.

Young children communicated their dental discomfort to parents, who could assist in managing the sensation.

with my mum, I'm like, ow (not ow, no). I'm like, mother, my teeth are sensitive. [laughter about using the word mother] I want to say mother; I don't want to say mummy. Nitro, male, aged 8 years

Older children seemed to be more reluctant to discuss their feelings of discomfort with parents, either because they felt misunderstood or to avoid subsequent dental treatment.

I looked on google, and then it came up with like, I need surgery and I need to take all my teeth out, so I never tell my mum. Because she would probably take me to the dentist. Gerald, aged 12 years

Although not formally invited to participate in the interviews, some parents expressed surprise that their children felt discomfort or adapted to TH as they had not shared these experiences with them previously.

### 3.5 Theme 5: Experience Within the Dental Context and Setting

Children described feeling TH during their dental appointments, discussed their dental anxiety and alluded to the burden of care related to them having MIH. Additionally, they discussed how the air syringe, dental probe, cold metal instruments, and suction could induce TH.

The air is meh. I don't like the air either. It's like, makes me feel really sensitive. Gerald, aged 12 years

Interestingly, children discussed that they felt TH when their family dentist blew cold air on the teeth to check if they were hypersensitive.

When the dentist blew it on. Oh, when the dentist blew cold air on it, yeah. To see how sensitive it was… Lucy, aged 8 years.

Referring to the dental probe:

The little scrappy thing…Yeah, that hurt, that's sharp. Gerald, aged 12 years

Older children occasionally used technical dental terms, revealing a degree of health literacy relating to MIH and TH:

I've got a condition called hypermineralisation and it's caused by lack of enamel in the teeth, which is caused by exposure to chicken pox during the time period from eight months pregnant to, is it 2 weeks old? Coco, aged 9 years.

When discussing going to the dentist, several children described anxiety.

It's cold like, it's dark and I can't see anything…[I feel] emotional and when I come to the dentist, my teeth feel up and down and just like really nervous. Butterfly, female aged 6 years

Whilst some children discussed that they felt more anxious about attending dental appointments when they were younger and didn't understand what the interventions would entail, others felt the opposite, reporting heightened apprehension during dental visits with increased encounters and understanding.

…when I was younger, they would just be shouting out random things, like random numbers…I didn't understand. But now I've been to the dentist more, I know, like, more about it…when they're on about: I think we should put this on them, or I think we should take them out, I feel a bit more, nerve wracking. Gerald, aged 12 years

Children discussed feeling anxious and seeking information online, often leading to misconceptions and fear of potential surgical interventions. However, they would not discuss these with their parents, as they felt it would lead to unwanted interventions. They reported feeling that the dentist and parents were acting against them.

Gerald: I was a bit scared…I was wondering what they were going to say…And my mum was on their side as well.

Interviewer: Do you feel that we're not on your side?

Gerald: Well, my side is like no pain, no surgery, no nothing.

Interviewer: And the dentist's side is…?

Gerald: Surgery, pain. (Gerald, aged 12 years).

Children also discussed the burden of care related to MIH.

I don't know specifically, but I've definitely been to the dentist a lot and I've had a lot of random different things. Maggie, aged 15 years

Finally, aspects of communication with the dentist were described by some children and young people. They also discussed helpful management strategies.

I mean maybe the composite bonding [would help], you know, because it adds a layer over the teeth…an extra barrier between the cold and my teeth. Maggie, aged 15 years

Giving them a little break for a second even if they don't ask, it does really help because some kids are a bit like me…a bit more nervous to ask. Maggie, aged 15 years

Emily played a song on their ukulele with the following lyrics:

I really like my teeth, you should really get a filling if you need it, it is very important to keep your mouth healthy, otherwise you're going to end up with no teeth. So, take my word, take my advice because you need to look after your teeth. Emily, aged 10 years

## 4 Discussion

This is the first purely qualitative study to explore children's experiences of TH in the context of MIH. As such, it provides a unique and rich insight into the everyday experiences and perspectives of young patients with this common dental condition. Unsurprisingly, many of the findings from the present study resonate closely with those from previous quantitative enquiries which have also involved children with MIH‐related TH [13]. Child‐reported generic OHRQoL measures have proven merit in capturing the negative functional, social, and emotional impacts experienced by larger cohorts of children with TH, as well as evidencing clinically meaningful change following tailored TH interventions [13, 14]. However, quantitative approaches, by their very nature, cannot provide us with new insights into how individuals behave and feel in both clinical and non‐clinical settings. We will now consider the key findings from our study, and the relevance they may have for clinical practice and further oral health research in this field.

Key findings and clinical/research implications: From the outset, it is fundamentally important that we use child‐appropriate language and descriptors to elicit a comprehensive MIH‐TH history to inform subsequent treatment planning. Findings from this study have revealed how children positioned themselves as experts in terms of their MIH‐TH and used a variety of concepts and words to communicate symptoms and impacts. They commonly used literal and sensory descriptions, such as “cold”, “hurt”, “sensitivity”, “sharp” and “tingling”. These terminologies share commonality with those used by adults with dentine hypersensitivity as well as words used by children with other dental or neuropathic conditions [15, 16, 17]. However, dentine sensitivity studies with adults found that patients tended to avoid the word “pain” and preferred the term “sensations”. This was not the case in our study where children referred to TH as “pain” or “hurting”. This reference to pain/hurting appears to be common in children's pain lexicon [16].

Of further interest was the fact that children described their TH both in terms of frequency (how often) and severity (how much). This contrasts with previous OHRQoL research where some investigators have reported that children showed a clear preference for severity descriptors [18]. Our findings therefore have implications for the validity of tools used to measure MIH‐TH in that they may need to consider both frequency and severity of impacts, due to the intermittent and transient nature of TH. Although studies with adults with dentine hypersensitivity have reported both frequency and intensity during the qualitative stage, the dentine hypersensitivity questionnaire does not explicitly address frequency [19]. In addition, asking children about the frequency and severity of any TH, it is also important to ask them about specific triggers. Findings from this study suggest that MIH‐TH was most commonly brought on by cold foods (specifically ice cream), cold drinks, cold air and dental interventions. Children identified certain healthy foods, such as cold fruit, as TH triggers. As these are generally considered important components of a balanced diet, avoidance linked to TH could have broader implications for children's nutritional status.

Traditionally, we may have given greatest credence to functional impacts within our history and diagnosis. However, this study has clearly highlighted the profound emotional aspects of MIH and TH. Children described weakness, frustration, confusion, and anxiety as well as feeling different from their peers. They struggled to understand why they experienced TH while others did not, especially when they were younger. Children described TH as different because it was “invisible,” differing from a more obvious pain aetiology such as a knee scratch. This invisible discomfort may be harder for others to acknowledge or value, potentially contributing to oral health stigma and influencing children's decision to seek help [20].

When asked about social impacts, children acknowledged that, on occasions, TH diminished their fun with friends. Interestingly, TH was described as a “looming fear”, which resonates with the hypervigilance reported by adults with dentine hypersensitivity [15]. This anticipatory fear of pain is likely to lead to heightened levels of anxiety in both social and dental settings, as well as increased pain sensitivity [21, 22].

Children described feelings of frustration about their parents not believing or understanding their TH experience, since it is not an obviously visible condition. On the other hand, children reported not disclosing their TH symptoms to parents, who were often surprised by their discussions of TH during our interviews. Non‐concordance between children and parents/carers in child OHRQoL research is not a new concept, but certainly one that brings additional challenges to clinical history‐taking [23].

Health literacy (HL) is now regarded as an integral facet of healthcare, although paediatric oral health literacy has not yet gained momentum within the field [24]. Put simply, health literacy is concerned with the acquisition and utilisation of knowledge relevant to an individual's health condition to better inform self‐care and decision‐making [25]. Admittedly, this study provided only a cursory insight into the HL needs of children with MIH‐TH, but it serves to highlight opportunities for more work in this area. An “acceptance” of TH was observed in older children, who discussed that getting older and having an increased understanding of TH and MIH allowed for better coping and acceptance. Furthermore, children reported brushing their teeth even if they experienced TH, showing not only awareness of the importance of oral hygiene but resilience in tolerating discomfort for a health benefit. Resilience mechanisms were noted to include perseverance with activities that bring health benefits to children with chronic pain [26].

Although some children reported symptom improvement following dental treatment, increased understanding of their condition, cognitive maturity, and improved emotion regulation also appeared to support acceptance of TH.

It would have been helpful to further explore how children sourced their information on MIH, and whether dental professionals had been instrumental in providing this. Child‐friendly and on‐line information specific to MIH is available, but this study did not specifically explore whether this had been accessed (https://www.thed3group.org/molar‐hypomin.html).

Children clearly described ways of managing their TH: distraction was one reported coping mechanism, with younger children discussing that TH would go away when playing with their toys and their siblings. Behavioural distraction (such as playing games) and cognitive distraction (such as thinking of something else) have been well described as passive pain coping mechanisms, alongside acceptance [27]. Asking for help from parents was mostly discussed by younger children. This concurs with findings from the wider paediatric pain literature where requests for parental help progressively decrease as children gain independence and may prefer to seek help from peers [28].

A lack of understanding about dental procedures and fear of the unknown were common concerns, again suggesting unmet oral HL needs in this population. Children expressed a desire for better communication and emphasised the importance of being proactive in advocating for their comfort during dental visits. This suggests that a greater awareness of how to communicate with and manage children with MIH‐TH is needed in specialist paediatric dentistry practice.

Implications for clinical practice: Children and adolescents may conceal or minimise pain to avoid judgement, reduce perceived social burden, or maintain normality, thereby reducing any risk of stigma, isolation, and diagnostic uncertainty [29]. Oral health stigma can lead to hiding concerns and prioritising visible problems over functional issues [30]. This highlights the need for an empathetic, child‐centred approach in which clinicians actively listen to the child's views and create an environment where they feel safe to share their concerns.

Inaccurate or misleading online information may add to treatment avoidance and under‐reporting of symptoms. Alongside fears arising from limited understanding of procedures, children described feeling overwhelmed or experiencing TH during dental appointments, but felt unable to speak up. Therefore, care should be tailored to take into account any reported individual triggers and adapting procedures, for example, by using warm water or avoiding cold air and ensuring adequate analgesia. Providing clear, age‐appropriate information can help reduce misinformation. Trust should be reinforced through simple explanations, agreeing stop signals and providing breaks. Care pathways should remain flexible, with feedback from children and parents guiding management.

Furthermore, TH should be recognised not only at an individual level but incorporated into wider care pathways. Clinicians should gain a contemporary and evidence‐based understanding of TH through targeted training and awareness‐raising initiatives, facilitating enhanced knowledge across different settings.

Reflections on the study design: Throughout this study, efforts were made to involve children meaningfully in keeping with accepted good practice [31]. As well as using children's own words, other means of communication were encouraged and acknowledged. Notably, children of all ages conveyed the feeling of TH with facial expressions, such as frowning, grimacing or shivering, in addition to verbal descriptions. Several children made sounds whilst grimacing or made the sign of putting their thumb down. A child played a song about MIH on their ukulele, with the lyrics also being included in the analysis. This is consistent with findings from other authors who have discussed that children may use non‐verbal communication extensively [32].

As the accuracy of children's pain recall is widely variable and depends on their cognitive development, it was felt that the onset and overall timeframe of pain should not be explored, due to the potential for introducing bias. Studies have reported a wide variation in the accuracy of pain intensity recall among children over the age of 7‐years‐old (55%–100%), with considerably lower consistency below this age (22%–55%). The overall accuracy between 5‐ and 15‐years‐old is 45% and remains at this value beyond 1 week recall [33, 34, 35].

In compliance with research ethics' protocol, parents were free to listen to their child's interview and provide support if needed. Although parental perspectives were not sought per se, when offered, they did provide some additional insights into children's experiences of TH within the broader family context. While parents were mostly silent during the interviews, their occasional interventions helped to clarify their child's explanations, terminologies, and recall about specific TH episodes. For younger children, this aligns with existing literature indicating that parental observations of visible signs may be more reliable than children's recall [36, 37]. Parents also expressed surprise at the extent of their child's experience with TH, which further highlighted the under‐recognition of this condition by families. It must however be acknowledged that parental presence during interviews, while providing valuable context, may also have influenced children's responses.

A main limitation of this study was the inclusion of children from a single specialist centre and who predominantly identified as having a White Northern European background. The lack of racial/ethnic diversity within the study group, despite a purposive sampling strategy, may therefore impact upon how representative our findings are as the vocabulary used and experiences described may be less culturally relevant to the wider population. Another possible limitation of our findings was the under‐representation of children with mild MIH. Children with mild MIH are more likely to be managed in primary care rather than being referred to tertiary centres. To overcome this potential issue, we aimed to recruit all children with MIH, irrespective of whether or not they reported TH symptoms at their initial assessment, thereby adding representational and inferential generalisability to this study.

It is also worth noting that the use of Wilson and Cleary's theoretical framework for the topic guide, while beneficial for providing structure, showed limitations when applied to children's experience of TH. The framework was developed for adults, and its domains often overlapped when mapping children's descriptions. As this framework did not fully capture the unique ways in which children described TH, thematic analysis was prioritised.

Future research directions: The Lancet Child and Adolescent Health Commission sets an agenda for paediatric pain, calling for action to make it matter, understood, visible, and better through recognition, research, improved assessment, and effective treatments [38]. Therefore, further research into MIH‐related TH in children is essential, including its cognitive, social, and cultural dimensions whilst considering children's cognitive and dental development.

An important piece of work would be to investigate in greater depth how children may emotionally, psychologically, or physiologically adapt to TH symptoms over time. Better understanding these adaptation processes could have direct clinical relevance for the development and evaluation of new management strategies.

Further research into the structural and biochemical basis of MIH related TH is necessary to inform effective interventions. Resources should also be directed towards research on treatment, including high‐quality randomised controlled trials using validated measures and child‐centred outcomes.

In line with Eccleston and colleagues' [38] recommendations, TH should be visible through correct diagnosis using patient‐reported outcome measures. Our first priority, therefore, will be to use the findings to inform the development and evaluation of a child MIH‐TH specific measure of OHRQoL. This tool will have significant benefit in terms of assessing children's presenting complaints, informing treatment decisions, and evaluating the effectiveness of any dental interventions in reducing TH‐related impacts. In this next phase, we will need to develop strategies to ensure there is wider participation of children from different racial/cultural backgrounds. Themes from the qualitative findings will be incorporated into the questionnaire, with children's own wording used to reflect their perspectives. Items will be modified iteratively following discussions with PPI groups, through cognitive interviews, as well as input from experts and parents, ensuring children's views remain central.

Although this was not an initial consideration, this study has identified an unmet HL need in our MIH patients and families. The study also revealed that some parents were unaware of their child's experiences with TH. There is therefore a great opportunity to explore ways to improve MIH‐related awareness and communication between children, parents, and healthcare professionals.

## 5 Conclusion

This is the first qualitative study to explore children's descriptions of MIH‐related TH and the negative impacts this may have on their everyday lives. Some valuable insights have been gained, which are relevant to clinicians and researchers, and these findings will now inform the development of an OHRQoL measure of TH specific to children with MIH.

## Author Contributions

J.M., F.G., H.R. conceived the ideas. J.M. collected the data. J.M., F.G., H.R. analysed the data and J.M. led the writing.

## Funding

This work was supported by the Royal College of Surgeons of England.

## Ethics Statement

Ethical approval was granted by the South Central‐Oxford C Research Ethics Committee (reference: 23/PR/1182, 13/11/2023).

## Conflicts of Interest

The authors declare no conflicts of interest.

## Data Availability

The data that support the findings of this study are available on request from the corresponding author. The data are not publicly available due to privacy or ethical restrictions.
